# Excess Body Weight and Long-Term Incidence of Lung and Colon Cancer in Men; Follow-Up Study of 43 Years

**DOI:** 10.3390/ijerph181910418

**Published:** 2021-10-03

**Authors:** Yftach Gepner, Shahar Lev-ari, Uri Goldbourt

**Affiliations:** 1 Department of Epidemiology and Preventive Medicine, Sackler Faculty of Medicine and Sylvan Adams Sports Institute, School of Public Health, Tel Aviv University, P.O. Box 39040, Tel Aviv 6997801, Israel; 2 Department of Health Promotion, Sackler Faculty of Medicine, School of Public Health, Tel-Aviv University, Tel-Aviv 39040, Israel; leva@tauex.tau.ac.il; 3 Department of Epidemiology and Preventive Medicine, Sackler Faculty of Medicine, School of Public Health and the Henry N. Neufeld Cardiac Research Institute, Tel-Aviv University, Tel-Aviv 39040, Israel; goldbu1@tauex.tau.ac.il

**Keywords:** lung cancer, colon cancer, cancer incidence, body mass index, cohort study

## Abstract

Most evidence for an association between excess body weight and cancer risk has been derived from studies of relatively short duration with little reference to the effect on tumor site. This study was designed to evaluate the association between categories of body mass index (BMI: <20, 20–25, 25–30, and >30 kg/m^2^) and the incidence of colon and lung cancer over 43 years of follow-up (1963–2006), in 10,043 men from the Israeli Ischemic Heart Disease (IIHD) prospective cohort (mean age at baseline 49.3 years, mean BMI 25.7 kg/m^2^). Data from the Israel National Cancer Registry was linked with the IIHD, and the Cox proportional hazards regression model was applied to analyze the relative risks for lung and colon cancer across BMI categories at baseline. Three hundred cases of lung cancer (2.9%) and 328 cases of colon cancer (3.3%) were diagnosed in the total population. Applying a multivariate model adjusted for age, smoking intensity, and total cholesterol, higher BMI category was associated with an increased risk of colon cancer [HR = 1.22 (95% CI 1.02–1.45)], and with a decreased risk for lung cancer [HR = 0.66 (95% CI 0.56–0.77)]. In this long-term follow-up study over four decades, we observed a consistent dose-response pattern between BMI and increased risk for colon cancer, but decreased risk for lung cancer. Specific associations between excess body weight and cancer risk may suggest different patterns of body fat and cancer incidence at a given site.

## 1. Introduction

Obesity is a progressively important health problem worldwide, which affects nearly one-third of the population in the industrialized world [1]. Several studies have shown that body mass index (BMI) is an independent risk factor for co-morbidities, including hypertension, stroke, cardiovascular disease, type 2 diabetes, and several types of cancers [2,3]. However, although several studies have reported that excess weight is an important predictor for risk and death from cancer [4,5,6,7,8], there is a paucity of information about how this relates to cancers at specific sites, or about the public health burden of excess weight in terms of cancer incidence.

Lung cancer is currently the second leading cause of cancer deaths in females and the leading cause among males globally [9], corresponding to 14% of cancer deaths in females and 24% in males [9]. Tobacco smoking is the main risk factor for lung cancer, and it accounts for 40% to 80% of lung-cancer deaths [10,11]. Paradoxically, some epidemiological studies have indicated an inverse relationship for BMI with lung-cancer risk [8,12,13], while others have reported a positive association [5,6].

Colon cancer, the fourth most common malignancy diagnosed, has been consistently linked with obesity [8], including a pooled analysis of seven prospective studies [14]. However, there are important limitations to evidence based on the relatively short-term follow-up studies. In addition, many studies have calculated BMI from self-reported weight and height data, which may underestimate the true value [15]. Thus, the diverse relationship between excess body weight and cancer risk at different sites remain debatable, particularly with respect to the association between BMI and the long-term development of lung and colon cancers.

Recently, the World Cancer Research Fund and American Institute for Cancer Research reports, state that there is strong evidence that body fatness increases the risk of colorectal cancer, and there is limited evidence between body fatness and lung cancer. In a large recent publication of incidence and mortality worldwide analysis for 36 cancers in 185 countries, it was found that the proportion of lung and colon cancers from all sites are 11.4% and 6%, respectively. Lung cancer remained the leading cause of cancer death (18%), and colon cancer contributes to 5.8% of total cancer deaths [16]. Therefore, the diverse relationships were found between and the highly prevalent cancers should be further tested in a long-term prospective study.

To gain a better understanding of the long-term relationship between excess body weight and colon and lung cancer risk, we conducted analyses of BMI and colon and lung cancer incidence based on the large-scale Israeli Ischemic Heart Disease study (IIHD). The purpose of the current analysis was to assess the relationship between excess body weight in men aged 40–65 and the risk to develop colon and lung cancer over four decades.

## 2. Materials and Methods

**Figure 1:**
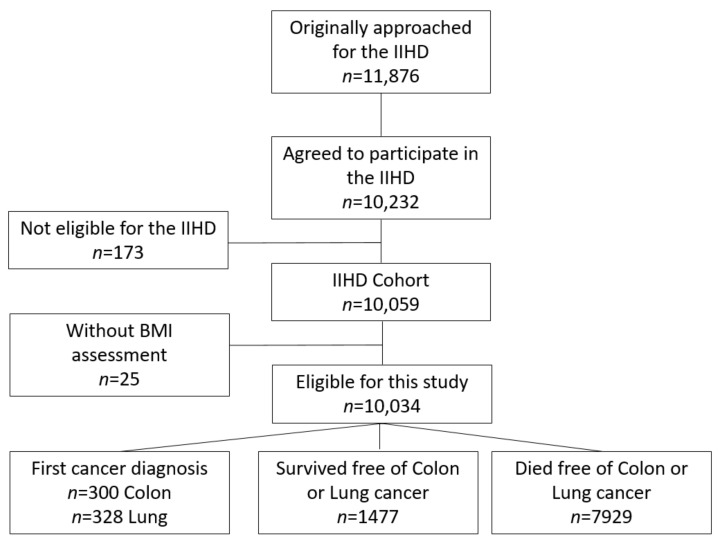
Flow chart of patients’ inclusion into the study.

This large-scale follow-up substudy, the Israeli Ischemic Heart Disease (IIHD) project, was first created in 1963. This cohort study (*n* = 10,232, 86% adherence) was comprised of tenured municipal employees and civil servants aged 40 years and above, selected by stratified sampling in 6 areas of birth (those born in Israel and those who were immigrants from 5 other prespecified areas). This cohort provided an extensive representation of the socioeconomic levels in the male working population of Israel at the time of inclusion [17]. Follow-up was restricted to participants (*n* = 10,059) who were born in one of six pre-defined geographic areas (Central Europe; Eastern Europe; the ‘Mideast’, Balkan countries; North Africa and Israel, Figure 1). Consenting participants were invited to an individual interview and requested to provide diverse socio-demographic and behavioral information by completing a psychosocial questionnaire including their dietary habits. In addition, an anthropometric assessment of weight, height, and blood pressure was recorded. Participants also underwent physical and electrocardiographic examinations and provided blood samples for bio-chemical evaluation. In the current study, survival time was based on a long-term follow-up of this population upon enrollment (1963) until the end of follow-up (31 December 2006), the date of death, or the date of lung or colon cancer diagnosis, whichever occurred earlier.

### 2.1. Body Mass Index Assessment

The calculation of BMI based on height without shoes, to the nearest centimeter, and weight to the nearest kilogram. BMI was calculated as the ratio of body weight in kilograms and squared body height in meters (kg/m^2^). Normal BMI was defined between 20 and 24.9 kg/m^2^, overweight as BMI between 25.0 and 29.9 kg/m^2^, and obese as BMI of 30.0 kg/m^2^ or higher [18].

### 2.2. Socioeconomic Status, Smoking Status and Physical Activity

The Socioeconomic Status Index (SES) (represented by a five-point index) was based on self-reported formal education and the type of employment. Education was divided into 9 levels, ranging from no formal schooling to graduate degree. Occupation comprised levels, ranging from ‘laborer’ to ‘professional’. A self-reported in-person questionnaire was used to determine smoking status using the classification of ever smoked (yes/no), or by category of number of cigarettes per day (never, quit, and 1–10, 11–20 or >20 cigarettes/day). Additional smoking status was evaluated at 1965 and for a subsample of 1053 participants at 1968. Change between smoking categories was noted between the three time points, and participants were classified into the following groups: increased, maintained, reduced, or quit smoking. Assessment of physical activity was performed in 1965, based on the following question: “what degree of leisure time physical activity do you practice?”. The answer that best described the degree of physical activity was to be chosen by the subject: (1) almost no physical activity, (2) sporadic physical activity, (3) daily physical activity, (4) daily intensive physical activity [19].

### 2.3. Lung and Colon Cancer Assessment

In 2012, the study cohort was cross referenced to the dataset of the Israel National Cancer Registry (INCR) in order to detect cancer cases that had occurred up to December 31, 2010. However, the analyses included only cancer cases diagnosed by 31 December 2006 because vitality status was updated only up to this time point. The INCR is a national, population-based passive registry, which was established in 1960. The cohort was linked with the database of the INCR at 2012 by the national identified number of the patient/participant. The INCR collects information on all in situ and invasive cancer cases as well as benign tumors of the brain and the central nervous system in Israeli citizens and its completeness is high (94% for solid tumors and 85% for hematopoietic tumors). Coding of tumors location and morphology followed the International Classification of Diseases for Oncology, Third Edition were used for lung cancer (C34) and colon cancer (C18).

### 2.4. Ethical Considerations

All participants had given their oral consent to take part in the study upon their recruitment in 1963 following explanations regarding the study objectives and the long-term follow up, before the existence of Ethical Review Boards in Israel. In addition, the Tel Aviv University Ethical Review Board approved the linkage between the IIHD database, the Israel Population Registry and the Israel National Cancer Registry in order to monitor the vital status of the participants and their cancer incidence.

### 2.5. Statistical Analysis

We used the Cox proportional hazards analysis in order to estimate hazard ratios (and 95% confidence intervals, CI) for lung and colon cancer incidence over time across BMI categories, compared to the category of normal BMI (BMI = 20–24.9 kg/m^2^). Age at diagnosis of cancer was used as the outcome. Multivariate model data were adjusted for age (five-year age groups), total cholesterol and cigarette smoking intensity at any time up to and including 1963 and 1965. Schoenfeld residuals tested in the proportional hazards assumption, and no violations were detected. A sensitivity analysis for lung cancer risk by different smoking variables (see Table 1) were performed using Cox models, we tested. All statistical analyses were preformed using STATA statistical package version 16.1 (STATA, College Station, TX).

## 3. Results

In total, 10,034 men who underwent height and weight measurements were eligible for the current analysis (Figure 1). The average age of the population at recruitment (1963) was 49.3 ± 6.8 years, mean SES score was 2.61 ± 1.23, mean BMI = 25.7 ± 0.33 kg/m^2^, total cholesterol = 210 ± 40 mg/dL, 68.5% were smokers, and 40.8% reported that they engaged in some type of physical activity. The baseline characteristics of the participants in this substudy, categorized according to BMI group, are presented in Table 1. During the 43 years of follow-up, there were 328 cases of lung cancer (3.3%) and 300 cases of colon cancer (2.9%) diagnosed in the study sample. Higher BMI categories were associated with elevated anthropometrics with respect to blood pressure, cardiometabolic markers, and physical activity (*p* < 0.05 for all). Reverse associations were found between the higher BMI categories and HDL cholesterol (*p* < 0.001) and smoking (*p* < 0.001).

**Figure 2:**
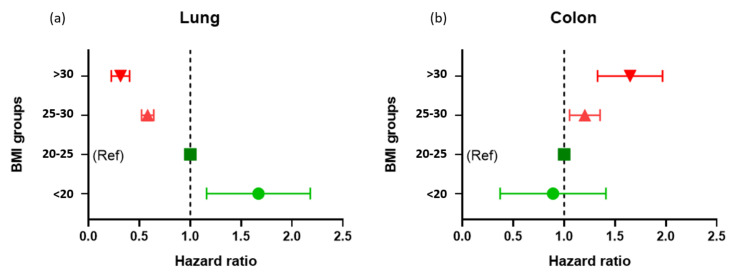
(**a**). Risk for lung cancer across groups of body mass index over 43 years of follow-up. Cox proportional hazards analysis with 95% confidence intervals for lung cancer incidence over time across BMI categories. The multivariate model was adjusted for age (five-year increments), height (cm), and cigarette smoking at any time up to and including 1963. (**b**). Risk for colon cancer across groups of body mass index over 43 years of follow-up. Cox proportional hazards analysis with 95% confidence intervals for colon cancer incidence over time across BMI categories. The multivariate model was adjusted for age (five-year increments), height (cm), and cigarette smoking at any time up to and including 1963.

Smoking was significantly associated with increased risk for lung cancer, but not for colon cancer. Analysis using a survival model adjusted for age revealed a significant risk for colon cancer [HR = 1.25 (95% CI 1.05–1.49)] with an increment of a single BMI category (<20, 20–24.9, 25–29.9, >30 kg/m^2^), while the risk for development of lung cancer decreased [HR = 0.55 (95% CI 0.46–0.66)]. Similar results for colon [HR = 1.22 (95% CI 1.02–1.45)] and lung cancer [HR = 0.66 (95% CI 0.56–0.77)] were found when the models were further adjusted for age, smoking (cigarette smoking intensity at any time up to and including 1963), and total cholesterol. Figure 2a,b present the multivariate hazard ratio for lung cancer according to BMI categories as compared to the recommended BMI category (20–24.9 kg/m^2^). The values for the dose-response relationship of increased risk for colon cancer with higher categories of BMI were as follows: <20 kg/m^2^ HR = 0.89 (95% CI = 0.28–2.80), 25–29.99 kg/m^2^ HR = 1.20 (95% CI = 0.94–1.53), and >30 kg/m^2^ HR = 1.64 (95% CI = 1.11–2.43). The values for the corresponding decreased risk for lung cancer with BMI category were <20 kg/m^2^ HR = 1.67 (95% CI = 0.90–3.07), 25–29.9 kg/m^2^ HR = 0.58 (95% CI = 0.46–0.73), and >30 kg/m^2^ HR = 0.32 (95% CI = 0.17–0.57). Next, in a sensitivity analysis Cox model, we tested the risk for lung cancer across BMI categories adjusted for different smoking variables. Compared to normal BMI category, similar trends were found when the model was adjusted for never smoked, smoked in the past and currently smoking (<20 kg/m^2^ HR = 1.44, 25–29.9 kg/m^2^ HR = 0.60, and >30 kg/m^2^ HR = 0.34) or for number of cigarettes per day, i.e., >20, 10–20 and 1–10 cigarettes/day plus nonsmoking group (<20 kg/m^2^ HR = 1.33, 25–29.9 kg/m^2^ HR = 0.64, and >30 kg/m^2^ HR = 0.36).

We further investigated the association between changes in smoking intensity from 1963 to 1965 among smoking participants and the risk for lung cancer. During this period, most of the participants (65%) maintained their smoking level, 27% reduced or quit smoking, and only 8% increased their cigarette consumption. When the Cox model was additionally adjusted for smoking intensity at 1965, an increment of a single BMI category (<20, 20–24.9, 25–29.9, >30 kg/m^2^) reduced the risk for lung cancer [HR = 0.658 (95% CI 0.560–0.774)], similarly to the smoking intensity at 1963. In a sensitive analysis of a subsample of 1053 participants that reported smoking status at 1968, lung cancer was also reversely associated with BMI [HR = 0.561 (95% CI 0.328–0.959)]. Same patterns were found for each BMI category with the risk of lung cancer between smoking status at 1963 and 1965.

Overall, the weight of the participants increased by 1.49 kg between 1963 and 1968. This corresponds to 0.53 BMI units, a change that was not associated with increased cancer risk during the follow up. Interestingly, the association between BMI and cancer incidence was very similar when measured based on smoking habits reported at 1963, 1965, and 1968, over the four decades of follow-up.

## 4. Discussion

In this long-term follow-up study over four decades, we observed a consistent dose-response profile between BMI and cancer incidence, although the outcome varied substantially for the cancer sites. A higher BMI was associated with an increased risk for colon cancer, but conversely, with a decreased risk for lung cancer. To our knowledge, this study is one of the longest large-scale studies to estimate the BMI–cancer association among men with tumors in different sites. This follow-up study of a working male population in Israel of marked ethnic, cultural, and occupational diversity represents immigrants from >20 countries on 3 continents, and therefore, it can be attributed to a wide population.

The results of this study are consistent with previous investigations that demonstrated a diverse association between obesity and the incidence of cancer at different sites. Colon cancer has been consistently associated with increased body weight and adiposity among men [8,20], in a variety of studies in the literature, including a pooled analysis of 8213 participants from 7 prospective studies [14]. Still, other studies reported that overweightness and obesity in adolescence can be associated with an increased risk of subsequent colon cancers in men and women [20]. The results of our study, adjusted for potential confounders, demonstrated that for middle-aged men (mean age of 49.3 ± 6.8 years), a high BMI is an independent risk factor for colon cancer in the long term (four decades). Furthermore, there was a strong dose response when those in the high BMI category (obese, BMI > 30) were compared to lean subjects (BMI < 25). This is in agreement with another large study that estimated the attributable risk of colon cancer to overweightness as 10.9% for European males and 2.6% for European females [21]. A meta-analysis of the effect of beneficial lifestyle factors on colon cancer incidence concluded that the risk ratios per 5 kg/m^2^ increase in BMI, varied between 1.16 and 1.28 for men, and between 1.04 and 1.12 for women [21]. However, another large study in Europe identified a BMI in the normal range as the leading lifestyle factor with the greatest benefits for colon cancer prevention [22]. Our data indicate that the attributable risk for an obese man (>30 kg/m^2^) was equivalent to 900 colon cancer cases per 100,000 person-years as compared to the normal weight group. Therefore, our study provides long-term evidence that weight reduction in individuals who are overweight or obese may be protective against colon cancer development.

In contrast, the results of our study revealed an inverse relationship between increase in body weight and the incidence of lung cancer. This was found even for obese subjects with BMI > 30. The lack of a biological explanation and weak or no association among nonsmokers have led some to suspect bias from residual confounding in smoking. Recent studies have demonstrated the higher BMI associated with better overall survival for some type of cancer, but the association was not consistent across cancer types and stages [23]. Lung cancer is the most common cause of cancer death worldwide, especially in developed countries [9]. Older age, cigarette smoking, African-American race, male gender, susceptible genotype, lower socioeconomic status, and low body weight have all been shown to increase the risk for developing lung cancer [24]. However, the inverse association between BMI and lung cancer concurs with other research [8,12,13]. More specifically, a recent meta-analysis found that low BMI category (<20) was a risk factor for lung cancer, while a BMI > 24.9 provided a potential protective effect against lung cancer [25]. In a recent study conducted over nine years, BMI was inversely associated with lung cancer risk regardless of stratification for sex, age, smoking status, alcohol drinking status, total physical activity, or menopausal status [22]. Moreover, a meta-analysis by Wang J et al., based on the results of 51 cohort studies, showed that increased BMI was significantly associated with improved survival of patients with lung cancer [26]. On the other hand, a number of studies failed to demonstrate this protective effect of overweightness or obesity on lung cancer [27], particularly among nonsmokers [5]. The vast majority of lung cancers occur among smokers, and the relatively strong inverse correlation we observed between BMI and the risk of lung cancer was adjusted for smoking status. Confounding by amount of smoking is the most likely explanation, while in our cohort, people with lower BMI tend to be heavy smokers (81.5% were ever smokers in the <20 BMI group as compared to 67.7% in the >30 BMI group). Those results has been reported in a previous large scale study [8]. However, we were unable to identify any other source of bias that could explain the association. Although it is still possible that the association between BMI and the risk of lung cancer is an artifact, the possibility of a distinct relationship between BMI and lung cancer among smokers should be considered. Future efforts to determine any other correlates of obesity that are associated with the risk of lung cancer could therefore be informative.

Our study has several potential limitations. (1) Weight and BMI data was only available at baseline (1963) and during the first phase of the follow up (1965 and 1968). (2) This cohort study was men only, and it is impossible to determine the association among women or to detect sex differences. This possibility limits our ability to generalize the results of an exclusively male population to women. (3) Our analyses were limited by the discrete recording of smoking status in 1963 and 1965, and we appreciate that changes in smoking status can change significantly during long-term follow-up. The lack of monitoring of smoking status in our study (starting of smoking or quitting smoking) may bias our findings on lung cancer risk. Additionally, the limited follow-up data regarding BMI and smoking intensity limit our analysis to determine interaction or mediation, but only association with cancer risk. Strengths of our study include the relatively large sample size and the long follow-up time of 43 years among a cohort of men who were extensively investigated for vascular risk and anthropometric factors, combined with the accuracy of detection of cancer provided by the Israel National Cancer Registry (INCR).

## 5. Conclusions

In conclusion, this long-term prospective study investigated the association between excess body weight and colon and lung cancer risk using data collected within the framework of the Israeli Ischemic Heart Disease cohort study. The results revealed that elevated BMI is associated with an increased risk for colon cancer, but with a decreased risk for lung cancer. These apparently paradoxical associations between excess body weight and cancer risk may reflect differences in the patterns of body fatness and cancer incidence in different sites. Further research will be needed in order to determine the mechanism responsible for our observed relationships between excess body weight and the incidence of lung and colon cancer and to verify our findings. We also suggest that further studies should determine other indexes associated with obesity, such as hip to waist ratio, with cancer risk at different sites.

## Figures and Tables

**Table 1:** Baseline characteristics of the Israeli Ischemic Heart Disease (IIHD) study population by body mass index group, *n* = 10,034.

	BMI Groups, kg/m^2^				
	<20 *n* = 146	20–24.9 *n* = 4079	25–29.9 *n* = 4863	>30 *n* = 946	*p*
**Demographic**					
Age (y)	48.2 ± 7.2	49.2 ± 6.9	49.4 ± 6.8	49.7 ± 6.7	0.07
Socioeconomic status	2.02 ± 1.2	2.52 ± 1.3	2.68 ± 1.2	2.37 ± 1.1	<0.001
**Anthropometric**					
Weight (kg)	49.1 ± 4.3	63.7 ± 6.9	75.9 ± 6.9	88.1 ± 7.2	<0.001
Height (cm)	166.6 ± 7.1	167.1 ± 6.7	167.3 ± 6.5	166.5 ± 6.4	0.27
**Blood pressure**					
Systolic Pressure (mmHg)	125 ± 17.9	132 ± 19.5	137 ± 20.8	143 ± 21.6	<0.001
Diastolic Pressure (mmHg)	78.1 ± 9.2	81.2 ± 10.3	85.3 ± 11.0	89.3 ± 12.3	<0.001
**Fasting blood biomarkers**					
Glucose (mg/dL)	83.1 ± 15.5	88.5 ± 26.8	89.8 ± 27.8	95.2 ± 35.1	0.003
Total cholesterol (mg/dL)	186 ± 34.8	203 ± 39.6	213 ± 39.7	214 ± 40.5	<0.001
HDL-c (mg/dL)	46.9 ± 11.4	42.3 ± 10.1	39.8 ± 9.1	38.8 ± 8.6	<0.001
Uric Acid	4.21 ± 0.83	4.47 ± 0.88	4.91 ± 0.94	5.24 ± 1.03	<0.001
**Lifestyle**					
Ever smoked, (%)	81.5	71.1	66.2	67.7	<0.001
Smoking status					<0.001
Never	17.6	30.2	34.3	32.6	
Quit	8.6	15.5	18.5	17.9	
1–10 cigarettes/day	14.5	14.4	15.2	17.4	
11–20 cigarettes/day	39.3	17.7	14.7	14.5	
>20 cigarettes/day	29.8	22.1	17.1	17.6	
Physical activity					0.025
None (%)	68.4	58.7	58.7	62.8	
Sporadic (%)	15.4	14.1	15.1	14.3	
Some (%)	10.3	19.1	18.8	15.1	
Intensive (%)	5.9	8.0	7.4	7.8	

Values in the Table are mean ± standard deviation.

## Data Availability

Data might be available upon direct communication with Goldbourt, an author in this publication, and the PI for the IIHD study database.
